# An Integrated Morphometric-Molecular Framework for Characterization of Developmental Stages in the Safflower Aphid *Uroleucon gobonis* (Matsumura)

**DOI:** 10.3390/ijms27177557

**Published:** 2026-08-24

**Authors:** Lanjie Xu, Sufang An, Yongliang Yu, Qing Yang, Zhansheng Nie, Huizhen Liang, Xiaohui Wu, Hongqi Yang, Junping Feng, Yazhou Liu

**Affiliations:** 1 Institute of Chinese Herbal Medicines, Henan Academy of Agricultural Sciences, Zhengzhou 450002, China; 2 Provincial Key Laboratory of Conservation and Utilization of Traditional Chinese Medicine Resources in Henan, Zhengzhou 450002, China

**Keywords:** *Uroleucon gobonis*, instar identification, morphological characterization, molecular markers, integrated pest management

## Abstract

Reliable identification of pest developmental stages provides a foundation for investigating aphid development and adaptive mechanisms and informs the selection of timely interventions in integrated pest management. In this study, eight morphological indicators of *Uroleucon gobonis* were distinguishing under a stereomicroscope. Additionally, six candidate genes identified from transcriptomic data were selected to characterize their expression profiles across five developmental instars. These results showed that the body length enabled distinguishing of the aphids from the first instar to the fourth instar, while antennal length and cauda length facilitated distinction between the second–fourth instar nymphs; cornicle length allowed separation of the third instar and subsequent instars. In contrast, body width permitted discrimination primarily between the first and second instars; head width, foreleg length, and hindleg length exhibited substantial overlap across instars, limiting their utility for instar discrimination. Elevated expression of *DN1031*, *DN1019*, and *DN1093* was a prominent feature of first instar nymphs. *DN1098* was persistently increased from the first to the third instar, which facilitated discrimination among these three developmental stages. The fourth instar was characterized by the concurrent down-regulation of *DN1098* and *DN1031* relative to the third instar, whereas adults exhibited a distinct expression peak of *DN136* compared to other developmental stages. This study establishes an integrated instar-identification system for *U. gobonis*, combining rapid morphological screening with molecular characterization. This framework provides a valuable methodological basis for investigating developmental plasticity and supports the development of targeted pest management strategies.

## 1. Introduction

Insect growth and development is a complex biological process that spans most of their entire life cycle. For agriculturally important pests, identifying population developmental stages provides a valuable basis for understanding growth patterns and life history strategies, and supports practical applications such as population monitoring, dynamics assessment, and optimizing of control strategies [1,2,3,4]. Among piercing- and sucking-mouthpart pests such as aphids, individuals at different developmental stages exhibit marked differences in feeding behavior, dispersal ability, reproductive potential, and tolerance to environmental stresses [5]. For example, young nymphs possess a thinner cuticle and are generally more susceptible to pesticides, representing a promising window for chemical control [2]. Therefore, developing efficient and reliable instar-identification techniques provides a valuable basis for investigating aphid developmental and adaptive mechanisms, advancing our understanding of pest population ecology, and informing the design of timely, targeted integrated management strategies.

Morphological identification is a commonly used method for determining aphid instars, assessing developmental stages through microscopic measurement or visual observation of external morphological traits [6]. This approach has been widely applied to differentiate adjacent instars. For example, instars of *Acyrthosiphon pisum* are often distinguished based on cornicle length [7], whereas those of *Sitobion avenae* are identified using cauda morphology [8]. Antenna length can serve as a key indicator for instar identification in *Megoura crassicauda* [9]. Although morphological traits can be influenced by environmental factors, traditional morphological methods remain widely used due to their operational simplicity, low technical requirements, and cost-effectiveness [10].

Molecular advances have provided potential molecular markers for insect instar discrimination. Insect development is associated with the spatiotemporally dynamic expression of genes. Accumulating evidence suggests that the expression levels of functional genes involved in key biological processes, such as molting and metamorphosis, often shift at specific developmental stages, contributing to distinct “gene expression fingerprints” [11,12,13]. In recent years, integrating transcriptomics to screen candidate marker genes with quantitative real-time polymerase chain reaction (*q*PCR) validation has emerged as a promising approach for developing molecular instar identification systems for insects [14]. However, systematic studies focusing on molecular instar identification in *U. gobonis* remain limited.

*Uroleucon gobonis* is a piercing–sucking pest that adversely affects economically important crops, particularly those in the family Asteraceae [15,16]. However, robust and practical methods for instar identification in this species are still limited. To address this gap, we assessed the discriminatory power of eight morphological traits across five developmental instars. Furthermore, six candidate genes were identified via transcriptome profiling and validated by *q*PCR to characterize their expression dynamics. This approach led to the development of an integrated “morphological prescreening–molecular verification” workflow, which supports reliable and efficient instar discrimination.

## 2. Results

### 2.1. Morphological Characteristics of U. gobonis Across Developmental Stages

**Figure 1:**
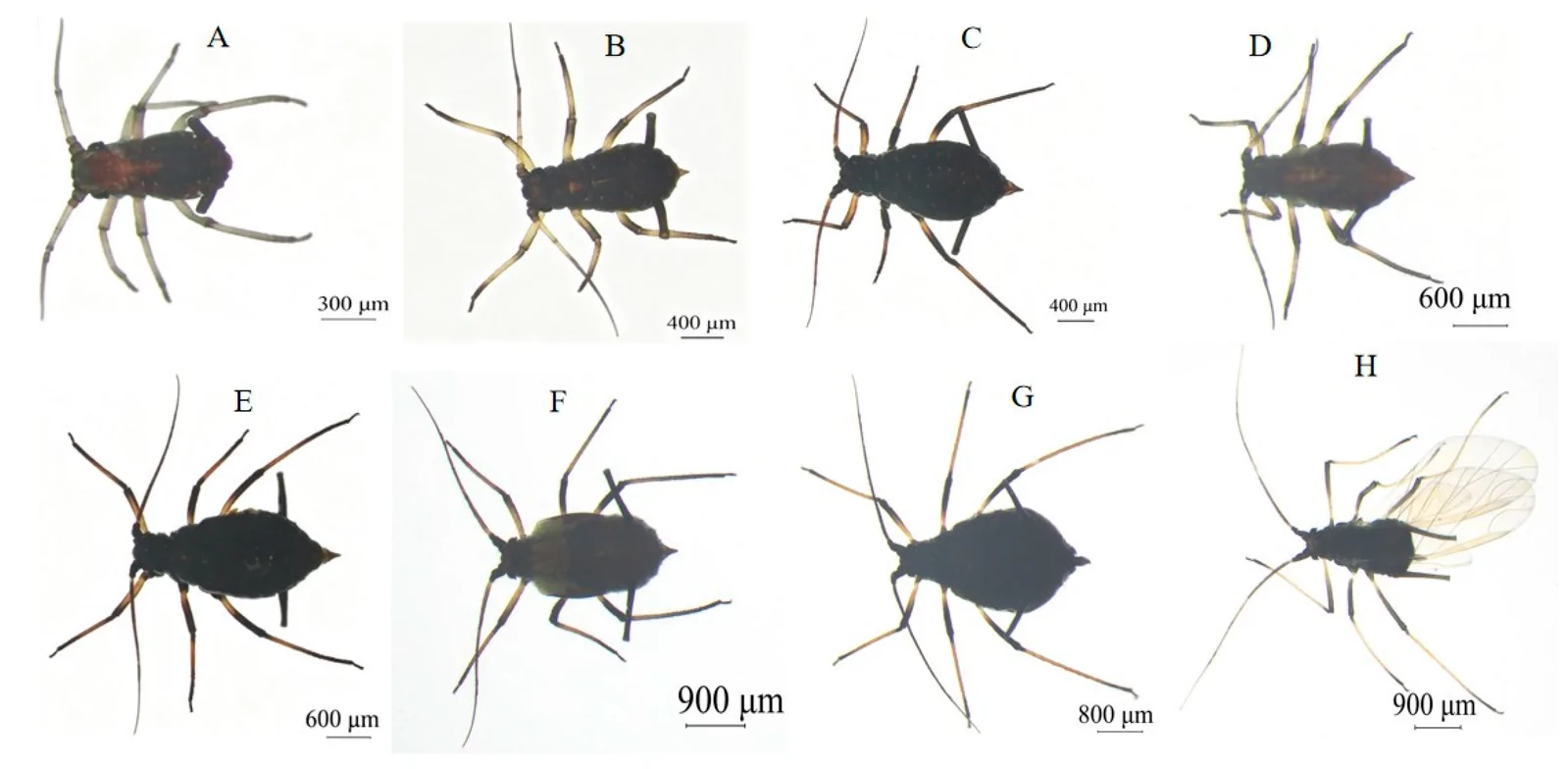
Representative morphological characteristics of *U. gobonis* at different developmental stages. (**A**) First instar nymph; (**B**) second instar nymph; (**C**) third instar nymph of apterous morph; (**D**) third instar nymph of alate morph; (**E**) fourth instar nymph of apterous morph; (**F**) fourth instar nymph of alate morph; (**G**) apterous adult; and (**H**) alate adult. Progressive development of body size, antennal length, and wing structures were evident across successive developmental stages.

*U. gobonis* undergoes five developmental stages, comprising four nymphal instars and the adult stage (Figure 1). Morphological characteristics of this species change progressively during development, offering useful diagnostic features for instar identification. The first instar and second instar nymphs share generally similar external morphology (Figure 1A,B), characterized by a relatively transparent body wall, comparatively short antennae, and rudimentary cornicles. Third instar nymphs exhibit clear morphological differentiation and can generally be categorized into two morphotypes based on wing base development: third instar apterous nymphs, which typically lack distinct protrusions on the mesonotum and wing buds (Figure 1C), and third instar alate nymphs, which display evident mesonotal protrusions and developing wing buds (Figure 1D). This differentiation provides a useful morphological indicator for distinguishing between second instar and third instar nymphs.

The third instar apterous nymphs retain a consistent meso- and meta-thoracic morphology during subsequent development, typically developing into fourth instar apterous nymphs (Figure 1E) and later into apterous adults (Figure 1G). By contrast, third instar alate nymphs show continued development of thoracic wing buds. By the fourth instar stage (Figure 1F), developing fore- and hind-wing buds become evident, and well-developed wing structures are present in adults (Figure 1H). This study demonstrates that the degree of wing base development serves as an important diagnostic feature for distinguishing between second instar nymphs and third instar alate nymphs, supporting further morphometric analysis.

### 2.2. Correlation Analysis of Instars and Morphological Indicators

All eight morphological indicators were significantly positively correlated with the five developmental stages of *U. gobonis* (*p* < 0.01). Among these, antennal length had the highest correlation coefficient with instars (r = 0.964), followed by body length (r = 0.960) and cornicle length (r = 0.943). Further analysis revealed a marked positive correlation (r = 0.979, *p* < 0.01) between antennal length and cornicle length, indicating that these two traits tend to increase in tandem during aphid development. The correlation coefficient between body width and body length was 0.960 (*p* < 0.01), consistent with a general scaling of body dimensions across developmental stages. Furthermore, head width, cauda length, and foreleg length were also strongly associated with developmental stages, with correlation coefficients exceeding 0.920 (Table 1).

### 2.3. Analysis of Morphological Indicators of U. gobonis Across Different Instars

Statistical comparisons of eight morphological traits were conducted based on mean values derived from 60 first instar nymphs, 60 s instar nymphs, and 30 alate and 30 apterous individuals each for the third instar, fourth instar, and adults. The results revealed that all eight morphological parameters generally increased with developmental stage, with body length, antennal length, and cornicle length displaying the relatively large increases compared to other traits. Antennal length rose approximately 2.9-fold from the first instar nymph to the adult stage, with a 20% increase from the second to third instar, a 15% rise from the third to fourth instar, and a 10% increase from the fourth instar to the adult stage. Statistically significant differences (*p* < 0.05) were detected between each pair of adjacent instars. Cornicle length expanded approximately 3.6-fold from the first instar nymphal stage to the adult stage, with a 25% expansion from the second to the third instars. Although the growth rate declined in subsequent stages, significant differences persisted across most developmental comparisons. Head width, hindleg length, and other measured morphological traits also increased significantly from the first instar to the adult stage (*p* < 0.05) (Table 2).

### 2.4. Overlap Analysis of Morphological Indicators Across Different Instars

**Figure 2:**
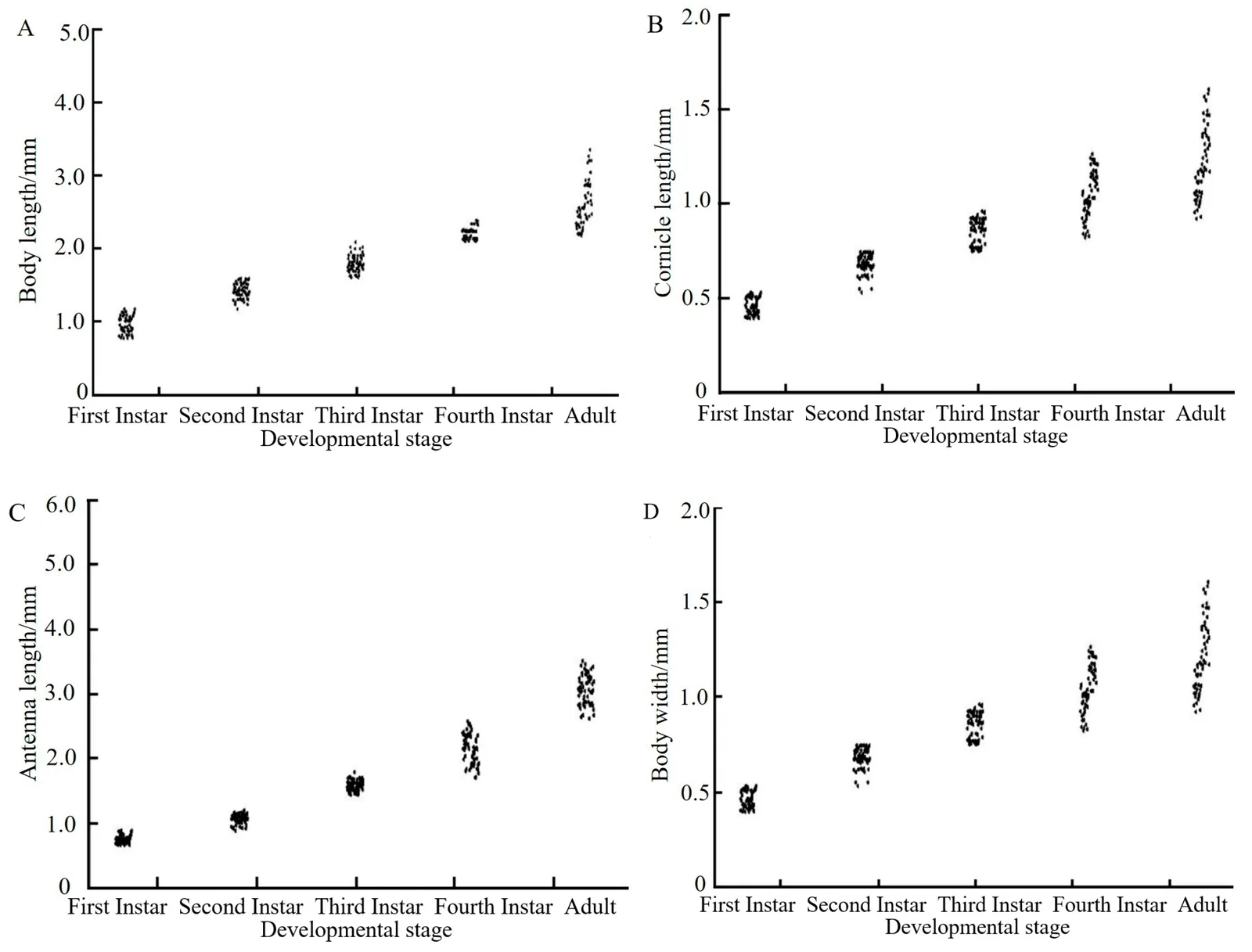

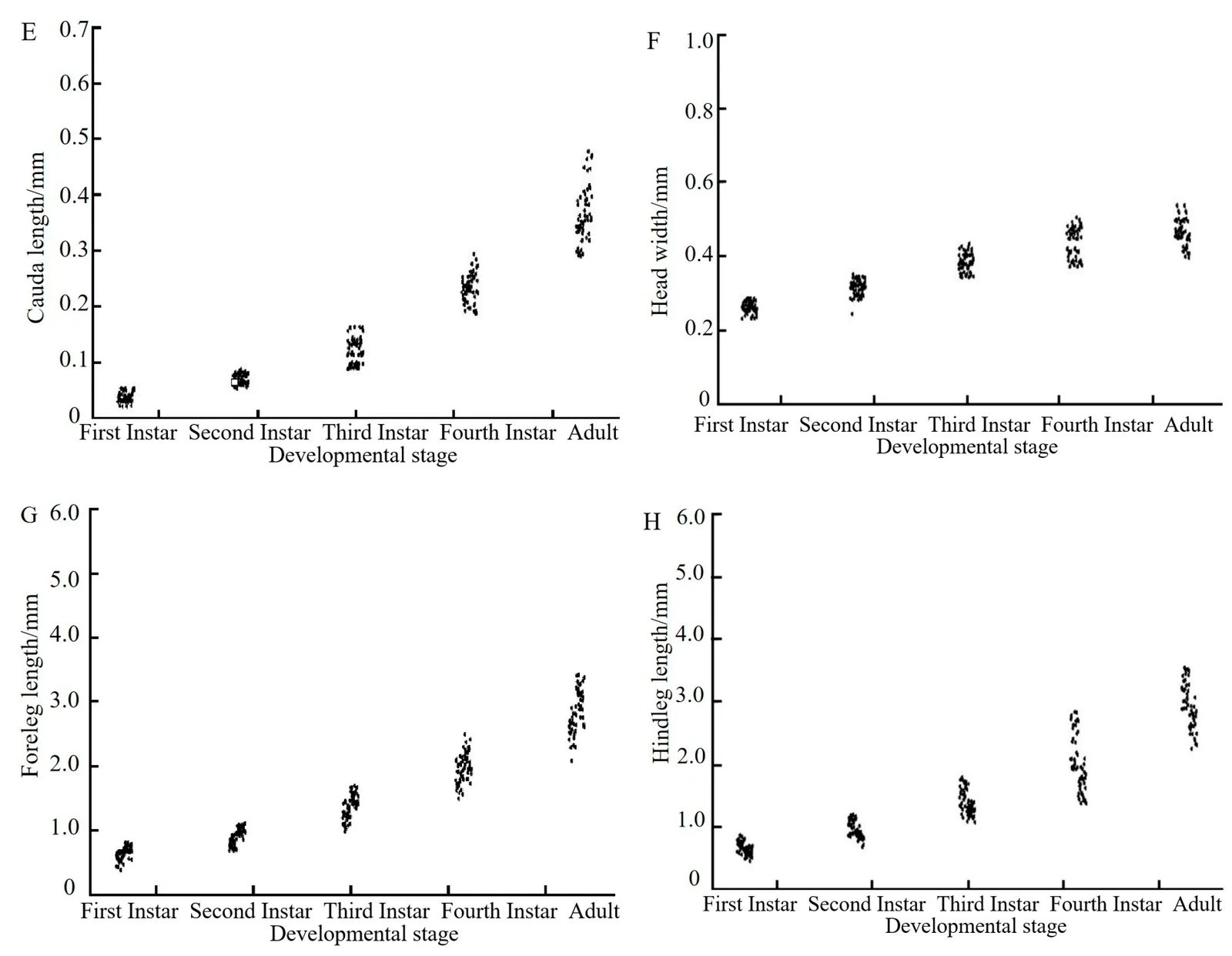
Distribution patterns of eight morphological traits of *U. gobonis* across different developmental stages. (**A**) Body length; (**B**) cornicle length; (**C**) antennal length; (**D**) body width; (**E**) cauda length; (**F**) head width; (**G**) foreleg length and (**H**) hindleg length. Each point represented an individual measurement, illustrating the degree of overlap among developmental stages and the diagnostic value of each trait for instar identification.

Based on measurements of eight morphological traits from 300 aphid specimens, box plot analysis revealed the following patterns of dispersion and central tendency across developmental stages (Figure 2). Body length can distinguish aphids from the first to the fourth instars. However, the maximum body length of fourth instar aphids (2.393 mm) was greater than the minimum observed body length of adults (2.181 mm), resulting in overlap between the body length ranges of these two developmental stages (Figure 2A). Cornicle length overlapped between the first and second instars (Figure 2B). However, starting from the third instar, the value ranges of each instar were completely separated, indicating that cornicle length reliably distinguished third instar, fourth instar, and adult aphid stages. Antennal length can distinguish aphids from the second to fourth instars (Figure 2C). Body width can distinguish between first and second instars (Figure 2D); however, substantial overlap occurred among subsequent developmental stages. Cauda length distinguished between second and third instar aphids and between third and fourth instars (Figure 2E); however, overlap occurred between first and second instars and between fourth instar aphids and adults. Head width (Figure 2F), foreleg length (Figure 2G) and hindleg length (Figure 2H) overlapped across all adjacent instars and therefore were not suitable as independent diagnostic traits for instar identification.

### 2.5. Expression Patterns of Six Genes Across Different Instars of U. gobonis

**Figure 3:**
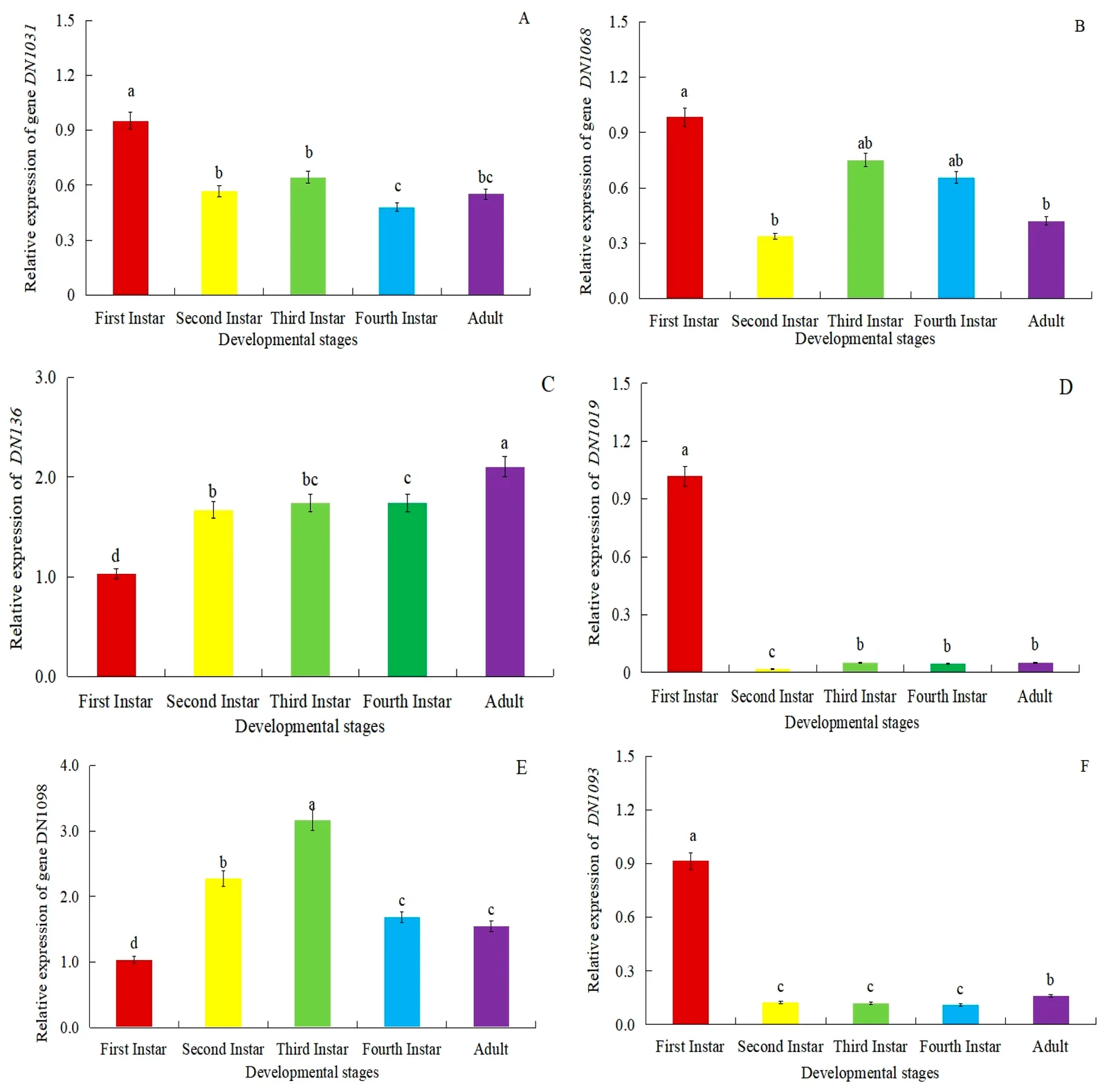
Expression patterns of candidate genes at different developmental stages of *U. gobonis.* (**A**) *DN1031*; (**B**) *DN1068*; (**C**) *DN136*; (**D**) *DN1019*; (**E**) *DN1098*; and (**F**) *DN1093*. Different lower-case letters above bars indicated significant differences among developmental stages for the same gene (*p* < 0.05). Error bars represented the standard error. Relative transcript abundance was determined by *q*PCR and normalized to the internal reference gene *L27*.

In this study, we analyzed the expression patterns of six candidate genes (*DN1031*, *DN136*, *DN1019*, *DN1068*, *DN1093*, and *DN1098*) across five developmental stages of *U. gobonis* using *q*PCR (Figure 3). The expression level of the *DN1098* gene showed a bell-shaped trajectory. Expression was lowest at the first instar aphid stage (d), increased significantly at the second instar (b), and displayed peak levels at the third instar (a); then, during the fourth instar and adult stages, expression declined to levels below those observed in the second instar (c). *DN1031* expression was highest at the first instar (a), decreased significantly in the second and third instars (b), and reached its lowest level in the fourth instar (c). *DN136* exhibited a gradual increase in expression, with the lowest level observed in the first instar (d); expression gradually increased with developmental stage and peaked in adults (a). *DN1019* was elevated in the first instar (a), decreased sharply to its lowest level in the second instar (c), and remained at relatively low levels in the third instar, fourth instar, and adult (b). *DN1068* expression was low in the first instar (c), increased significantly in the second instar (b), remained at peak levels during the third and fourth instars (a), and decreased slightly in adults (ab). *DN1093* was highly expressed in the first instar (a) and significantly reduced in the second and third instar and adult stages (b).

In summary, the first instar was associated with elevated expression of *DN1031*, *DN1019*, and *DN1093*. The transition from the first to second instars coincided with up-regulation of *DN1098* and an increase in *DN1068*. Sustained upregulation of *DN1098* aids in distinguishing the second, third, and fourth instars. The transition from the third to the fourth instars involved the concurrent downregulation of *DN1098* and *DN1031*, whereas adults exhibited peak expression of *DN136*.

## 3. Discussion

Accurate identification of pest developmental stages offers a valuable basis for studies of developmental ecology and for determining favorable timing for pest management interventions [17,18]. In this study, we developed an integrated approach combining morphological and molecular methods for instar identification in the agriculturally important pest *U. gobonis*.

Morphometrics represents a conventional approach for insect instar identification and has been widely adopted in this field owing to its operational simplicity and low cost [19,20,21]. We first assessed significant differences in the mean values of eight morphological traits of *Uroleucon gobonis* across five developmental stage, followed by an evaluation of the correlations between developmental stages and these traits. The results revealed that all eight morphological parameters increase, progressing with advancing instar, with body length, antennal length, and cornicle length exhibiting the largest increments; moreover, all eight indicators were significantly and positively correlated with developmental stages (*p* < 0.01), which is consistent with previous findings for closely related aphid species such as *Acyrthosiphon pisum* and *Aphis gossypii* [7,22,23]. Additionally, box plot analyses were conducted based on measurements from 300 aphid specimens. The results revealed that developmental stages were significantly correlated with all eight traits, with mean values differing significantly among the five developmental stages. However, the analysis also indicated that, after excluding traits exhibiting inter-instar range overlap, body length aided in distinguishing first–fourth instar nymphs. Cornicle length helped differentiate aphids from the third instar onwards, antennal length and cauda length showed utility in separating second–fourth instar nymphs, and body width allowed discrimination between first and second instar nymphs. In contrast, head width, foreleg length, and hindleg length provided limited diagnostic utility for instar determination.

To explore the potential of molecular methods for aphid instar determination, six candidate genes were identified via transcriptomic analysis, and their expression dynamics across five developmental stages were characterized by *q*PCR. Notably, *DN1031*, *DN1019*, and *DN1093* exhibited consistently elevated expression in first instar nymphs (fold change > 8.0; *p* < 0.01), which may reflect their involvement in the critical transition from embryo to nymph [24,25]. *DN1031* encodes a solute carrier family 2 member protein; homologs in other insects have been implicated in the transmembrane transport of monosaccharides, a process that could support the rapid growth and development of early instar nymphs [26,27]. *DN1019* encodes a vacuolar protein sorting-associated protein 13, which may function in intracellular trafficking material transport and vesicle sorting. *DN1093* encodes a ZBED8-like protein, a putative transcriptional regulator that might affect developmental transitions via modulation of downstream target gene networks. The concurrent high expression of these three genes constitutes a notable molecular feature of first instar nymphs, although their direct involvement in early cuticle formation and activation require further investigation in *U. gobonis*. *DN1098* exhibited peak expression in third instar nymphs (fold change = 12.7; *p* < 0.001), consistent with a possible contribution to wing morph differentiation and reproductive development [28,29,30]. Similar stage-specific molecular markers have been reported in model insects such as *Drosophila melanogaster* [31]. *DN136* expression peaked in adults (fold change = 9.3; *p* < 0.01), suggesting its potential as an adult-associated molecular marker. *DN1068* maintained elevated expression during the second to fourth nymphal instars and may be involved in growth and developmental processes during the intermediate and late nymphal stages [32,33,34].

Overall, we propose a step-wise framework for *U. gobonis* instar classification. Importantly, because the gene expression profiles were determined via relative quantification, they do not provide absolute numerical thresholds for standalone diagnostics. Therefore, classification of unknown samples should follow an integrated approach:

(1) Primary morphological screening: Initial categorization should rely on morphometrics. For example, body length effectively separates early (first/second) from late (third/fourth) instars, and antennal length differentiates the second from the fourth instar.
(2) Targeted molecular confirmation: Molecular markers are deployed specifically to resolve morphological ambiguities (e.g., due to specimen damage or overlapping traits). It is not necessary to assay all six genes. For instance, high relative expression of DN1031 and DN1019 can confirm first-instar status; DN1098 profiling can distinguish the third instar (peak expression) from the fourth instar (concurrent down-regulation of DN1098 and DN1031); and DN136 expression acts as a marker for adults.
(3) Handling ambiguities: If intermediate gene expression values are observed, it suggests a transitional molting phase. In such scenarios, instar assignment should fall back to non-overlapping morphological traits (e.g., cornicle length), with molecular markers serving as supplementary indicators of developmental progression. Future studies employing absolute quantification (e.g., digital PCR) will be required to establish strict diagnostic thresholds for field applications.”

## 4. Materials and Methods

### 4.1. Aphid Rearing

The *U. gobonis* aphids used in this study were collected from the Henan Modern Agricultural Development base and maintained in the laboratory for multiple generations on safflower (*Carthamus tinctorius*) seedlings in an artificial climate chamber (Model RDN-300, Ningbo Yanghui Instrument Co., Ltd., Ningbo, China). Chamber conditions were set to 16 ± 2 °C, 45 ± 5% relative humidity, and a 16 L:8 D photoperiod. Seedlings were refreshed every 30 days.

Safflower leaves with intact petioles were excised and placed in 10 cm × 10 cm plastic dishes, with petioles inserted into moist absorbent cotton. A single apterous adult was transferred to each leaf using a soft brush. After the onset of nymphiparity, newly hatched nymphs were isolated and transferred to fresh dishes. Molting was monitored daily, and individuals at each nymphal instar, as well as adults, were collected for subsequent analyses.

### 4.2. Sampling for Morphometric Measurement

Morphometric parameters were measured for a total of 300 aphids using stereomicroscope. Samples were grouped as follows: 60 first instar nymphs and 60 s instar nymphs; for the third instar, fourth instar, and adults, 30 apterous and 30 alate individuals were included for each stage. Mean values for each trait were calculated from individual measurements and used for statistical comparisons. This sampling method differed from that used for the gene expression analysis.

### 4.3. Sampling for qPCR Gene Expression Analysis

The number of aphids per biological replicate was defined as follows: 10 individuals for first and second instar nymphs, and 5 individuals for each wing morph (apterous/alate) of the third instar, fourth instar, and adult stages. In each replicate, aphids of the same instar and wing morph were pooled and homogenized prior to total RNA extraction. The three biological replicates were prepared separately, with aphid collections, RNA extractions, and reverse transcriptions performed in separately experimental runs.

During *q*PCR amplification, three technical replicates were performed for the cDNA template from each biological replicate. For statistical analysis, the mean of the three technical replicates was calculated for each biological replicate. Differential expression analyses were conducted using only the means of the three biological replicates; technical replicates were not considered as independent biological samples.

### 4.4. Measurement of Morphological Traits

**Figure 4:**
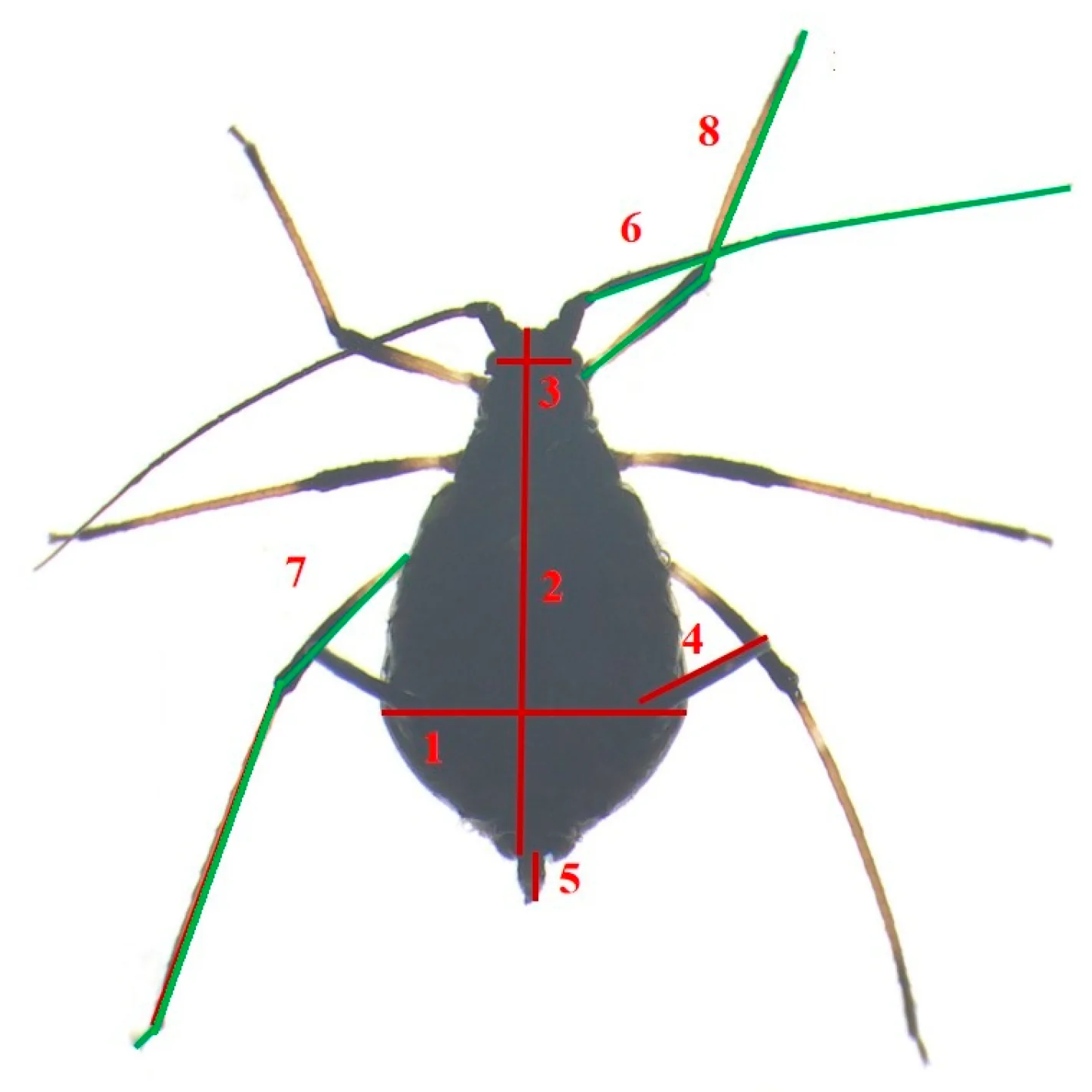
Morphometric traits and measurement locations of *U. gobonis.* 1: body width; 2: body length; 3: head width; 4: cornicle length; 5: cauda length; 6: antennal length; 7: hindleg length; and 8: foreleg length. Numbers indicate the anatomical landmarks used for morphometric measurements.

Each aphid was placed individually into a transparent glass dish containing 1 mL of 75% ethanol. Morphological traits of individual aphids were subsequently measured using a stereomicroscope (Model SZX7; Olympus Corporation, Tokyo, Japan). The eight morphological measurement indices are illustrated in Figure 4. For each specimen, each morphological trait was measured in three independent replicates, and the average value was incorporated into subsequent statistical analyses.

### 4.5. Candidate Gene Screening and Validation

Candidate genes were initially identified from previously generated transcriptomic datasets. To ensure significant and robust stage-specific expression, the following quantitative thresholds were applied: (i) absolute log_2_ fold change (|log_2_FC|) ≥ 1 between at least two adjacent developmental stages; (ii) false discovery rate (FDR) < 0.05; and (iii) minimum expression level ≥ 5.0 FPKM in at least one developmental stage to exclude transcripts with extremely low abundance that might compromise qPCR reliability. Additionally, genes exhibiting high variance among biological replicates within the same developmental stage were removed.

A total of 15 candidate genes met these criteria. Subsequent experimental validation was performed using *q*PCR to assess technical variability and reproducibility. Nine of these candidates were excluded due to high intra-group variability or because their *q*PCR expression profiles lacked consistency with the transcriptomic trends. Ultimately, six genes (*DN1031*, *DN1068*, *DN1093*, *DN1019*, *DN136*, and *DN1098*) exhibiting stable, stage-specific, and experimentally robust expression patterns were retained for comprehensive downstream analysis.

### 4.6. Total RNA Extraction and Real-Time Fluorescence Quantitative PCR Analysis

Total RNA was extracted from the samples using the Quick RNA Isolation Kit (Code No: Hyytb220; Beijing Huayueyang Biotechnology Co., Ltd., Beijing, China) following the manufacturer’s instructions. The quality and integrity of the total RNA were evaluated using 1.2% (*w*/*v*) agarose gel electrophoresis, and the RNA concentration was determined using a NanoDrop 2000 spectrophotometer (Thermo Fisher Scientific, Waltham, MA, USA). First-strand cDNA was synthesized from total RNA using the PrimeScript™ RT Reagent Kit with gDNA Eraser (Code No. RR047A; Takara Bio Inc., Kusatsu, Shiga, Japan) following the manufacturer’s protocol.

*q*PCR was used to assess gene expression profiles of six genes across five developmental stages of *U. gobonis*. Primers were designed using Primer Premier 5.0 (Table 3), and *L27* was used as the internal reference gene [35,36]. Amplification were performed with TaKaRa TB Green^®^ Premix Ex Taq™ II (Code No. RR820A; Takara Bio Inc., Kusatsu, Shiga, Japan). The thermal profile included initial denaturation at 95 °C for 3 min, followed by 45 cycles (95 °C for 10 s, 55 °C for 30 s, and 72 °C for 28 s). Specificity was checked via melt-curve analysis (55 °C to 94 °C, 0.1 °C/s). Each sample was run in three technical replicates. Relative expression was calculated using the 2^−ΔΔCT^ method. Statistical analyses employed one-way ANOVA with Tukey’s HSD post hoc test (*p* < 0.05). Data are shown as mean ± SE; *n* = 3 (biological replicates).

### 4.7. Data Analysis

All data were analyzed using Excel 2021 and SPSS 19.0. One-way ANOVA was performed, with subsequent multiple comparisons conducted using Duncan’s test (*p* < 0.05), to compare morphological traits across developmental stages of *U. gobonis.* Pearson correlation analysis was used to examine associations between morphological traits and developmental stages. Box plots were employed to visualize the extent of overlap in traits between adjacent instars. Traits were considered diagnostic traits when the overserved ranges showed no overlap between adjacent developmental stages., whereas traits with overlap were considered unsuitable for independent instar discrimination.

## 5. Conclusions

This study developed an integrated morphological–molecular approach for identifying the developmental instars of *U. gobonis*. Morphologically, body length aided in distinguishing first–fourth instars; cauda length and antennal length helped differentiate second–fourth instars; cornicle length supported the separation of third instar and subsequent instars. At the molecular level, elevated expression of *DN1031*, *DN1019*, and *DN1093* was associated with first instar nymphs; the gradual upregulation of *DN1098* from the first to the third instar contributed to discriminating among these early stages; and fourth-instar nymphs were distinguished by the concurrent down-regulation of *DN1098* and *DN1031* relative to the third instar. Adults exhibited a marked expression peak of *DN136*, a pattern that may guide the future design of adult-biased molecular markers. Collectively, these findings provide a framework for exploring developmental plasticity and adaptive evolution in aphids and may assist in refining integrated pest management strategies targeting *U. gobonis*.

## Figures and Tables

**Table 1:** Correlation analysis between developmental stages and morphological indicators of *U. gobonis*. ** indicate statistical significance at *p* < 0.01.

Indicators	Instar	Body Length	Body Width	Head Width	Cornicle Length	Cauda Length	Antenna Length	Foreleg Length	Hindleg Length
Instar	1.000								
Body length	0.960 **	1.000							
Body width	0.927 **	0.960 **	1.000						
Head width	0.927 **	0.893 **	0.869 **	1.000					
Cornicle length	0.943 **	0.905 **	0.847 **	0.879 **	1.000				
Cauda length	0.942 **	0.933 **	0.908 **	0.856 **	0.929 **	1.000			
Antenna length	0.964 **	0.937 **	0.892 **	0.888 **	0.979 **	0.962 **	1.000		
Foreleg length	0.947 **	0.938 **	0.928 **	0.869 **	0.931 **	0.967 **	0.968 **	1.000	
Hindleg length	0.924 **	0.876 **	0.810 **	0.861 **	0.965 **	0.917 **	0.953 **	0.892 **	1.000

**Table 2:** Morphological indicators of *U. gobonis* at different developmental stages. SE: standard error. Values were presented as mean ± SE and range. Different lowercase letters within the same row indicate significant differences (*p* < 0.05) according to Duncan’s multiple range test among developmental stages for the same morphometric trait.

External Morphometric Traits (mm)		Developmental Stages				
		First Instar	Second Instar	Third Instar	Fourth Instar	Adult
Body length	Mean ± SE	0.982 ± 0.015 a	1.430 ± 0.013 b	1.803 ± 0.015 c	2.208 ± 0.010 d	2.580 ± 0.039 e
	Range	0.779–1.178	1.178–1.596	1.610–2.093	2.107–2.393	2.181–3.360
Body width	Mean ± SE	0.465 ± 0.006 a	0.681 ± 0.06 b	0.859 ± 0.009 c	1.058 ± 0.016 d	1.211 ± 0.024 e
	Range	0.397–0.533	0.533–0.747	0.753–0.964	0.825–1.267	0.924–1.610
Head width	Mean ± SE	0.261 ± 0.002 a	0.317 ± 0.003 b	0.386 ± 0.003 c	0.434 ± 0.005 d	0.468 ± 0.005 e
	Range	0.232–0.287	0.245–0.352	0.344–0.436	0.373–0.507	0.397–0.540
Cornicle length	Mean ± SE	0.170 ± 0.002 a	0.282 ± 0.004 b	0.398 ± 0.004 c	0.633 ± 0.016 d	0.854 ± 0.013 e
	Range	0.142–0.198	0.198–0.356	0.298–0.447	0.458–0.851	0.654–1.039
Cauda length	Mean ± SE	0.038 ± 0.001 a	0.070 ± 0.001 b	0.122 ± 0.003 c	0.234 ± 0.003 d	0.366 ± 0.006 e
	Range	0.022–0.054	0.054–0.088	0.089–0.164	0.188–0.295	0.291–0.479
Antenna length	Mean ± SE	0.755 ± 0.007 a	1.074 ± 0.009 b	1.593 ± 0.011 c	2.148 ± 0.031 d	3.086 ± 0.030 e
	Range	0.667–0.887	0.887–1.204	1.446–1.797	1.712–2.584	2.627–3.520
Foreleg length	Mean ± SE	0.648 ± 0.013 a	0.908 ± 0.016 b	1.373 ± 0.024 c	1.983 ± 0.029 d	2.852 ± 0.043 e
	Range	0.383–0.812	0.690–1.115	0.989–1.696	1.498–2.497	2.088–3.428
Hindleg length	Mean ± SE	0.659 ± 0.013 a	0.954 ± 0.017 b	1.396 ± 0.027 c	2.006 ± 0.057 d	2.943 ± 0.046 e
	Range	0.455–0.868	0.679–1.200	1.075–1.964	1.378–2.844	2.254–3.557

**Table 3:** Primer sequences used for *q*PCR.

Gene Name	Forward Primer (5′–3′)	Reverse Primer (5′–3′)
*DN1031*	AACACAGGACACGCCTGATT	CAATTTCGTCGTTGGCCTGG
*DN1068*	TGGCATCGTTATCCAAACCCA	CACCAATGTCTTTTTGGTGGGA
*DN1093*	ATTTGACCAACGGGCGAAAC	TAATGTGGCCGACCATAGCC
*DN1098*	TAGCCCCGTGTTGGTCAAAG	AACAGATTCCTCGCCACCAG
*DN1019*	AGCTAGCTTTGTACCAGCGG	AGCATGTAGCCAAAACGTTGA
*DN136*	CTTCCGTCTTCGTCCTGCAT	AAATGACGCGGGCTACTCAA
*L27*	CCGAAAAGTGTCATAATGAAGACC	GGTGAAACCTTGTCTACTGTTACATCTTG

## Data Availability

The original contributions presented in this study are included in the article. Further inquiries can be directed to the corresponding authors.

## References

[1] Klingenberg C.P. (2010). Evolution and development of shape: Integrating quantitative approaches. Nat. Rev. Genet..

[2] Simon J.C., d’Alençon E., Guy E., Jacquin-Joly E., Jaquiéry J., Nouhaud P., Peccoud J., Sugio A., Streiff R. (2015). Genomics of adaptation to host-plants in herbivorous insects. Brief. Funct. Genom..

[3] Roy S., Saha T.T., Zou Z., Raikhel A.S. (2018). Regulatory pathways controlling female insect reproduction. Annu. Rev. Entomol..

[4] Pedigo L.P., Rice M.E., Krell R.K. (2021). Entomology and Pest Management.

[5] Dixon A.F.G. (2012). Aphid Ecology an Optimization Approach.

[6] Lü J., Wang L., Zhang K., Li D., Gao M., Guo L., Tang Z., Gao X., Zhu X., Wang L. (2024). Morphological characteristics, developmental dynamics, and gene temporal expressions across various development stages of *Aphis gossypii* sexual female. J. Cotton Res..

[7] Zhao H.Z., Yang Y., Zhang J.L., Li J.J., Zhao C.D., Shi Y., Liu T.X. (2021). Morphological characteristics for distinguishing the instars of *Acyrthosiphon pisum*. Chin. J. Appl. Entomol..

[8] Xu X.L., Liu X.X., Zhang Q.W., Wu J.X. (2014). Identification of the instars of *Sitobion avenae* (Fabricius) (Hemiptera: Aphididae). Acta Entomol. Sin..

[9] Yuan Y., Zhang T.W., Shi L., Xin J.Y. (2025). Morphological identification of different instars of *Megoura crassicauda*. Chin. J. Appl. Entomol..

[10] Abdullah H.M., Mohana N.T., Khan B.M., Ahmed S.M., Hossain M., Islam K.H.S., Redoy M.H., Ferdush J., Bhuiyan M.A.H.B., Hossain M.M. (2023). Present and future scopes and challenges of plant pest and disease (P&D) monitoring: Remote sensing, image processing, and artificial intelligence perspectives. Remote Sens. Appl. Soc. Environ..

[11] Merzendorfer H., Zimoch L. (2003). Chitin metabolism in insects: Structure, function and regulation of chitin synthases and chitinases. J. Exp. Biol..

[12] Riddiford L.M. (2012). How does juvenile hormone control insect metamorphosis and reproduction?. Gen. Comp. Endocrinol..

[13] Zhang C.X., Brisson J.A., Xu H.J. (2019). Molecular Mechanisms of Wing Polymorphism in Insects. Annu. Rev. Entomol..

[14] Xue J., Zhou X., Zhang C.X., Yu L.L., Fan H.W., Wang Z., Xu H.J., Xi Y., Zhu Z.R., Zhou W.W. (2014). Genomes of the rice pest brown planthopper and its endosymbionts reveal complex complementary contributions for host adaptation. Genome Biol..

[15] Abbot P., Tooker J., Lawson S.P. (2018). Chemical Ecology and Sociality in Aphids: Opportunities and Directions. J. Chem. Ecol..

[16] Xu L.J., Yu Y.L., Yang H.Q., Tan Z.W., Dong W., Li L., Li C.M., Liang H.Z. (2021). Screening of Safflower Germplasm with Resistance to *Uroleucon gobonis* and Field Efficacy Experiment. J. Nucl. Agric. Sci..

[17] Chen Q., Li N., Wang X., Ma L., Huang J.B., Huang G.H. (2017). Age-stage, two-sex life table of *Parapoynx crisonalis* (Lepidoptera: Pyralidae) at different temperatures. PLoS ONE.

[18] Reineke A., Thiéry D. (2016). Grapevine insect pests and their natural enemies in the age of global warming. J. Pest Sci..

[19] Daly H.V. (1985). Insect morphometrics. Annu. Rev. Entomol..

[20] García-Barros E. (2015). Multivariate indices as estimates of dry body weight for comparative study of body size in Lepidoptera. Nota Lepidopterol..

[21] Luo J.Y., Xie Q. (2024). Advances of major technology in insect morphology. J. Environ. Entomol..

[22] Ma N.W., Xia S.K., Liu B., Hu W.H., Wang P.L., Lu Y.H. (2025). Parasitism of different species and instars of cotton aphids by *Binodoxys communis*. Chin. J. Biol. Control.

[23] Xu L.J., An S.F., Yu Y.L., Yang Q., Tan Z.W., Li C.M., Su X.Y., Sun Y., Liang H.Z. (2024). Distinguishment of the Instars of *Macrosiphoniella yomogicola* in *Artemisia argyi*. J. Henan Agric. Sci..

[24] Karasawa T., Koshikawa S. (2025). Evolution of gene regulatory networks in insects. Curr. Opin. Insect Sci..

[25] Jacobs C.G., Rezende G.L., Lamers G.E., van der Zee M. (2013). The extraembryonic serosa protects the insect egg against desiccation. Proc. R. Soc. B Biol. Sci..

[26] Liu K., Dong Y., Huang Y., Rasgon J.L., Agre P. (2013). Impact of trehalose transporter knockdown on *Anopheles gambiae* stress adaptation and susceptibility to *Plasmodium falciparum* infection. Proc. Natl. Acad. Sci. USA.

[27] Behm C.A. (1997). The role of trehalose in the physiology of nematodes. Int. J. Parasitol..

[28] Ogawa K., Miura T. (2014). Aphid polyphenisms: Trans-generational developmental regulation through viviparity. Front. Physiol..

[29] Vellichirammal N.N., Gupta P., Hall T.A., Brisson J.A. (2017). Ecdysone signaling underlies the pea aphid transgenerational wing polyphenism. Proc. Natl. Acad. Sci. USA.

[30] McStay B. (2016). Nucleolar organizer regions: Genomic ‘dark matter’ requiring illumination. Genes Dev..

[31] Katoh H., Harada A., Mori K., Negishi M. (2002). Socius is a novel Rnd GTPase-interacting protein involved in disassembly of actin stress fibers. Mol. Cell. Biol..

[32] Anholt R.R.H., O’Grady P., Wolfner M.F., Harbison S.T. (2020). Evolution of Reproductive Behavior. Genetics.

[33] Herranz R., Mateos J., Mas J.A., García-Zaragoza E., Cervera M., Marco R. (2005). The coevolution of insect muscle TpnT and TpnI gene isoforms. Mol. Biol. Evol..

[34] Nijhout H.F., Callier V. (2015). Developmental mechanisms of body size and wing-body scaling in insects. Annu. Rev. Entomol..

[35] Mutti N.S., Park Y., Reese J.C., Reek G.R. (2006). RNAi knockdown of a salivary transcript leading to lethality in the pea aphid, *Acyrthosiphon pisum*. J. Insect Sci..

[36] Kang Z.W., Liu F.H., Tian H.G., Zhang M., Guo S.S., Liu T.X. (2017). Evaluation of the reference genes for expression analysis using quantitative real-time polymerase chain reaction in the green peach aphid, *Myzus persicae*. Insect Sci..

